# Lung Cancer Screening by Race and Ethnicity in an Integrated Health System in Hawaii

**DOI:** 10.1001/jamanetworkopen.2021.44381

**Published:** 2022-01-20

**Authors:** Caryn E. S. Oshiro, Timothy B. Frankland, Joanne Mor, Carmen P. Wong, Yannica Theda Martinez, Cheryl K. K. Aruga, Stacey Honda

**Affiliations:** 1 Kaiser Permanente Hawaii, Center for Integrated Health Care Research Honolulu, Hawaii; 2 Kaiser Permanente Hawaii, Hawaii Permanente Medical Group Honolulu, Honolulu, Hawaii

## Abstract

**Importance:**

Racial and ethnic differences in lung cancer screening (LCS) completion and follow-up may be associated with lung cancer incidence and mortality rates among high-risk populations. Aggregation of Asian American, Native Hawaiian, and Pacific Islander racial and ethnic groups may mask the true underlying disparities in screening uptake and diagnostic follow-up, creating barriers for targeted, preventive health care.

**Objective:**

To examine racial and ethnic differences in LCS completion and follow-up rates in a multiethnic population.

**Design, Setting, and Participants:**

This population-based cohort study was conducted at a health maintenance organization in Hawaii. LCS program participants were identified using electronic medical records from January 1, 2015, to December 31, 2019. Study eligibility requirements included being aged 55 to 79 years, a 30 pack-year smoking history, a current smoker or having quit within the past 15 years, at least 5 years past any lung cancer diagnosis and treatment, and cancer free. Data analysis was performed from June 2019 to October 2020.

**Exposure:**

Eligible for LCS.

**Main Outcomes and Measures:**

Screening rates were analyzed by self-reported race and ethnicity and completion of a low-dose computed tomography (LDCT) test. Diagnostic follow-up results were based on the Lung Imaging Reporting and Data System (Lung-RADS) staging system.

**Results:**

A total of 1030 eligible LCS program members had an order placed; their mean (SD) age was 65.5 (5.8) years, and 633 (61%) were men. The largest racial and ethnic groups were non-Hispanic White (381 participants [37.0%]), Native Hawaiian or part Native Hawaiian (186 participants [18.1%]), and Japanese (146 participants [14.2%]). Men and Filipino, Chinese, Japanese, and non-Hispanic White individuals had a higher proportion of screen orders for LDCT compared with women and individuals of the other racial and ethnic groups. The overall LCS completion rate was 81% (838 participants). There was a 14% to 15% screening completion rate gap among groups. Asian individuals had the highest screening completion rate (266 participants [86%]) followed by Native Hawaiian (149 participants [80%]) and non-Hispanic White individuals (305 participants [80%]), Pacific Islander (50 participants [79%]) individuals, and individuals of other racial and ethnic groups (68 participants [77%]). Within Asian subgroups, Korean (31 participants [94%]) and Japanese (129 participants [88%]) individuals had the highest completion rates followed by Chinese individuals (28 participants [82%]) and Filipino individuals (78 participants [79%]). Of the 54 participants with Lung-RADS stage 3 disease, 93% (50 participants) completed a 6-month surveillance LDCT test; of 37 individuals with Lung-RADS stage 4 disease, 35 (97%) were followed-up for additional procedures.

**Conclusions and Relevance:**

This cohort study found racial and ethnic disparities in LCS completion rates after disaggregation of Native Hawaiian, Pacific Islander, and Asian individuals and their subgroups. These findings suggest that future research is needed to understand factors that may be associated with LCS completion and follow-up behaviors among these racial and ethnic groups.

## Key Points

**Question:**

Are there racial and ethnic disparities in lung cancer screening (LCS) completion and diagnostic follow-up rates among Asian, Native Hawaiian, and Pacific Islander individuals?

**Findings:**

In this cohort study of 1030 adults in Hawaii, 838 (81%) completed LCS. There was a 14% to 15% screening completion rate gap between the Korean individuals (94%) and the Filipino (79%), non-Hispanic White (80%), and Pacific Islander groups (79%), although the differences were not significant.

**Meaning:**

These findings suggest that LCS completion rates may vary among Asian individuals and their subgroups, Native Hawaiian individuals, and Pacific Islander individuals, further highlighting the need to disaggregate these heterogenous groups to adequately understand specific factors that may be associated with LCS program participation.

## Teaser

This cohort study examines racial and ethnic differences in lung cancer screening completion and follow-up rates in a multiethnic population in Hawaii.

## Introduction

Lung cancer is the leading cause of cancer deaths in both men and women in the US.^1^ As of this writing, the American Cancer Society estimates that there will be approximately 235 760 new lung cancer cases and 131 880 lung cancer deaths in 2021.^2^ In the 2016 Cancer Facts and Figures report,^3^ a special section on cancer incidence and mortality among Asian American, Native Hawaiian, and Pacific Islander (AANHPI) individuals highlighted the disproportional lung cancer incidence and mortality rates that exist among these racial and ethnic groups. Moreover, these differences may not be immediately apparent because cancer data on AANHPI individuals in the US has generally been presented in aggregate, masking differences in the cancer burden that exists within racial and ethnic subpopulations.^3,4^ Lung cancer is the leading cause of cancer-related mortality among men of all Asian American ethnicities and of Chinese, Filipino, and Japanese women.^5^ Chinese women (in Asia and the US) have high lung cancer rates even though the prevalence of smoking in this group is low.^3^ In Hawaii, lung cancer is among the most common cancers,^6^ with the highest lung cancer incidence in Native Hawaiian men and women (79.9 and 64.4 cases per 100 000, respectively) and Filipino men (71.7 cases per 100 000).^7^ The findings of these studies further emphasize the importance of disaggregating AANHPI racial and ethnic groups and by sex to tease apart true differences that may exist in lung cancer rates.

Lung cancer screening (LCS) and smoking behaviors also vary by race and ethnicity and may explain differences in incidence rates. Samoan men have a 30% higher rate of lung cancer than Native Hawaiian, non-Hispanic White, and Laotian men and a 80% higher rate than Asian Indian and Pakistani men, which may be attributed to differences in smoking prevalence.^8^ Another study^9^ reported that Native Hawaiian and African American smokers were more likely to receive a diagnosis of lung cancer than smokers of any other racial or ethnic group. In 2013, the US Preventive Services Task Force recommended that adults aged 55 to 80 years, with a 30-pack year history of smoking and a current smoker or who had quit in the last 15 years be screened for lung cancer using low-dose computed tomography (LDCT).^10^ Since the implementation of these guidelines, studies have reported an overall low LCS uptake of less than 5% (proportion of eligible individuals who underwent screening) among populations that were predominantly non-Hispanic White and with limited data on AANHPI individuals.^11,12,13^ In a recent study^14^ featuring similar population proportions of screen-eligible non-Hispanic White individuals (43%) and African American individuals (41%) in the Boston Medical Center lung screening program, the LCS uptake rate was 16.1%. However, African American individuals had a lower screen rate of 37.6% compared with a screen rate of 46% among non-Hispanic White individuals.^14^ In a closer look at the LCS process within health care systems, few studies report on LCS completion rates (ie, the proportion of eligible individuals who completed a test after an order was placed), where variability in clinician orders may exist^15^ and completion may be dependent on inclusion of shared decision-making.^16^ Follow-up rates to a positive screen have also been reported,^17^ but few studies have examined both LCS completion and follow-up rates among men and women, Asian subgroups, and Native Hawaiian and Pacific Islander racial and ethnic groups.

This study examined LCS completion and follow-up rates among men and women, Asian subgroups, Native Hawaiian individuals, and Pacific Islander individuals participating in an LCS program within a safety-net health care system using a Nurse Navigator system. We also report overall diagnostic outcomes for this multiethnic population.

## Methods

### Study Design and Population

This is a population-based cohort study that examined Kaiser Permanente Hawaii (KPHI) electronic medical record (EMR) data of members who met the KPHI LCS program criteria from January 1, 2015, to December 31, 2019. Study protocols and human participants’ considerations were reviewed and approved by the institutional review boards at KPHI and Kaiser Permanente Colorado, which is the institutional review board of record for Lung Cancer Screening Optimization in the US (LOTUS, A Lung PROSPR II Research Center^18^). A waiver of informed consent was requested from the KPHI institutional review board and was approved on August 6, 2018, because this study posed no more than minimal risk to participants. This study follows the Strengthening the Reporting of Observational Studies in Epidemiology (STROBE) reporting guideline.

### LCS Program Eligibility and Completion Rates

The KPHI LCS program eligibility criteria included members who were aged 55 to 79 years, had a 30 pack-year smoking history, were current smokers or had quit within the past 15 years, were at least 5 years past any lung cancer diagnosis or treatment, and were cancer free. The most important risk factor associated with lung cancer is smoking, and it is estimated to account for approximately 80% to 90% of lung cancer cases.^19^ KPHI uses an Advanced Practice Registered Nurse Navigator to accurately assess smoking history and administer activities for the LCS program. This includes screening clinician-referred patients to ensure eligibility, providing shared decision-making discussion for program participation and smoking cessation, ordering and evaluating LDCT tests, and maintaining communication and clinical reports that track LCS participants through the program. We compared KPHI EMR data with the Nurse Navigator clinical reports to enhance our understanding of the underlying data and identify discrepancies. The Nurse Navigator provided LCS program clinical guidance on screening patterns that emerged through EMR data, such as specific time intervals used for follow-up.

We considered an LDCT order a proxy for both being screen eligible and agreeing to participate in the LCS program. *Current Procedural Terminology* code G0297 was used to identify screening orders within a 91-day window (orders to expiration date is 90 days) and to subsequently ascertain test completion and corresponding dates. Screening completion rates were calculated as the number of members with an LDCT completion date divided by the total number of members who had a screening order placed. For this study, eligibility criteria included clinician-referred with LDCT test ordered within 1 year of referral and before a second clinician referral into the LCS program, self-reported race and ethnicity information available in the EMR, and LDCT completion.

### Screening Follow-up and Diagnostic Outcomes

The Lung Imaging Reporting and Data System (Lung-RADS) version 1.0 is used by clinical practitioners as a standardized quality assurance tool to classify LDCT results and facilitate outcome monitoring.^20^ Lung-RADS version 1.0 categorization (stage 1, 2, 3, or 4 disease) was abstracted from the radiology report. Lung-RADS 4 subcategories (stages 4A, 4B, and 4X) are not used at KPHI, and we mainly focused on patients whose disease was staged at 3 and 4 to examine follow-up completion and diagnostic outcomes. Lung-RADS version 1.1 was adopted in 2019; however, there were minimal changes to classification of nodule size specific to stages 2 and 3. A 6-month surveillance LDCT is recommended for follow-up of Lung-RADS stage 3 findings.^21^ We selected a 5- to 12-month window to allow for capture of 6-month surveillance LDCTs for scheduling practicalities, which could occur at any time around 6 months. Follow-up for Lung-RADS stage 4 disease included EMR codes indicating a pulmonary consultation and orders for procedures including biopsy or other noninvasive procedures (eg, positron emission tomography scan), anytime from 0 to 12 months after the original screen date. EMR review was performed to validate and confirm follow-up completion and diagnostic outcomes that were based on surgical biopsy report, categorized as non–small cell carcinoma, small cell carcinoma, nonepithelial, and benign. Other aspects of the biopsy were not captured (eg, specific type).

### Demographic Variables

Age at referral for screening and sex were available from the EMR. Age was categorized into 5-year age brackets except for the 70 to 74 years and 75 to 79 years categories, which were collapsed to protect patient identification. Self-reported race and ethnicity are gathered at the time of KPHI membership enrollment and are available in the EMR. Racial and ethnic categories included were Asian (Chinese, Filipino, Japanese, and Korean), Hispanic, Native Hawaiian or part–Native Hawaiian, non-Hispanic White, other Pacific Islander or part–other Pacific Islander, and other (African American, American Indian or Alaska Native, and other Asian).

KPHI does not collect individual socioeconomic data. For a measure of socioeconomic status, we used the member’s address at the time of LCS program referral to ascertain their US Census tract and associated neighborhood deprivation index (NDI). The NDI is a *z* score that is generated through a principal component analysis from US Census tract variables in socioeconomic and demographic domains that characterize the lack of resources that a particular Census tract may experience.^22^ These domains include education, employment, housing, occupation, poverty, residential stability, and racial composition. NDI mean (SD) values are presented by race and ethnicity, where a higher score represents more neighborhood deprivation (a lack of resources).

### Statistical Analysis

Continuous variables are expressed as means (SDs), and categorical variables are expressed as frequencies (number and percentages). We used Pearson χ^2^ test to test for associations between racial and ethnic groups, sex, and screening completion and follow-up rates. Statistical procedures were conducted using SAS statistical software version 9.4 (SAS Institute), and significance was set at 2-sided *P* < .05. Data analysis was performed from June 2019 to October 2020.

## Results

The EMR data waterfall of 838 KPHI members eligible for the LCS program from referral to completion of an LDCT is shown in Table 1. We compared the demographic characteristics of 862 patients who did not have an LDCT order placed in the required time frame and thus were lost to follow-up with the 1030 patients who did have a LDCT order placed. We did not find any significant differences across age, sex, or race and ethnicity, so we do not have evidence that these 2 groups of patients are different. In addition, we also compared the age and sex of those who were missing race and ethnicity data (80 participants) with those with race and ethnicity data (1812 participants) and did not find any significant differences between these groups.

**Table 1.** Electronic Medical Record Waterfall of Kaiser Permanente Hawaii Lung Cancer Screen Referred Population to Low-Dose Computed Tomography Completion

Criteria	Participants, cumulative No.
Total referrals in the electronic medical record	2982
Total unique patient records	1975
Age eligible	1892
Order placed anytime	1138
Order placed within 1 y of referral date	1106
No second referral date before order placement date	1072
With race and ethnicity data	1030
Completed low-dose computed tomography	
With race and ethnicity data	855
Within 91 d of order date with race and ethnicity data	838

Table 2 highlights the 1030 LCS program participants who had an order placed and demonstrates the diversity of racial and ethnic categories, including Asian subgroups (Chinese, Japanese, Korean, and Filipino), Native Hawaiian, non-Hispanic White, Pacific Islander, and other participants. The largest racial and ethnic groups were non-Hispanic White (381 participants [37.0%]), Native Hawaiian or part Native Hawaiian (186 participants [18.1%]), and Japanese (146 participants [14.2%]). Eligible LCS program members had a mean (SD) age of 65.5 (5.8) years, and 633 (61%) were men. Screen eligible age is presented in 2 categories (55-64 years and >65 years) because of small sample sizes in the older age categories. However, after separating participants into 5-year categories by racial and ethnic groups, we observed that the highest proportion of Native Hawaiian, Pacific Islander, and Filipino individuals were present in the younger age group (60-64 years), whereas the highest proportion of Asian subgroups, non-Hispanic White individuals, and other individuals appeared in the next age category of 65 to 69 years. Mean (SD) NDI values by racial and ethnic group ranged from −0.51 (0.43) for Korean individuals to −0.07 (0.60) for Filipino individuals (higher NDI values represent higher levels of neighborhood deprivation).

**Table 2.** Racial and Ethnic Categories of Lung Cancer Screen Eligible Program Members by Demographic Characteristics

Characteristic	Participants, No. (%) (N = 1030)							
	Chinese	Filipino	Japanese	Korean	Non-Hispanic White	Native Hawaiian or part Native Hawaiian	Other Pacific Islander or part Pacific Islander	Other^a^
Total	34 (3.3)	99 (9.6)	146 (14.2)	33 (3.2)	381 (37.0)	186 (18.1)	63 (6.1)	88 (8.5)
Sex								
Male	27 (79.4)	76 (76.8)	97 (66.4)	15 (45.5)	242 (63.5)	90 (48.4)	37 (58.7)	49 (55.7)
Female	7 (20.6)	23 (23.2)	49 (33.6)	18 (54.6)	139 (36.5)	96 (51.6)	26 (41.3)	39 (44.3)
Age, y								
55-64	15 (44.1)	41 (41.4)	54 (37.0)	14 (42.4)	157 (41.2)	87 (46.8)	37 (58.7)	40 (45.5)
>65	19 (55.9)	58 (58.6)	92 (63.0)	19 (57.6)	224 (58.8)	99 (53.2)	26 (41.3)	48 (54.5)
Neighborhood deprivation index, mean (SD)^b^	–0.44 (0.47)	–0.07 (0.60)	–0.47 (0.47)	–0.52 (0.42)	–0.42 (0.50)	0.27 (0.66)	–0.26 (0.63)	–0.39 (0.54)

^a^ The other race and ethnicity category includes other Asian, African American, and American Indian or Alaska Native individuals.

^b^ Increasing values of the deprivation index represent increased levels of neighborhood deprivation.

**Figure 1.**
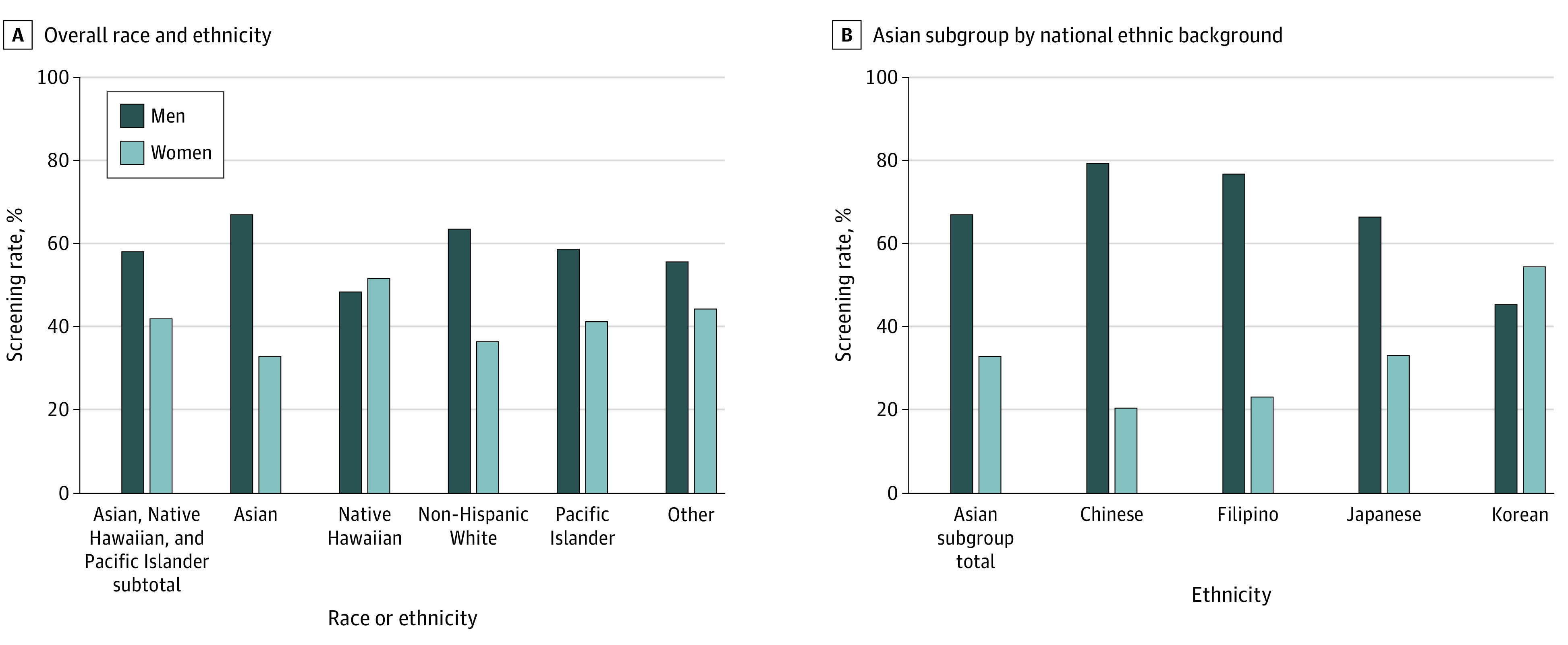
Low-Dose Computed Tomography Orders by Race and Ethnicity and Sex The other race and ethnicity category includes other Asian, African American, and American Indian or Alaska Native individuals.

A higher proportion of men (1142 participants [60%]) than women (750 participants [40%]) were clinician referred and age eligible for the KPHI LCS program (total, 1892 participants) (Table 1). However, this difference was not significant. There were differences in the sex distribution of LDCT orders (a proxy for eligibility LCS program confirmation) between racial and ethnic groups (χ^2^_7_ = 35.05; *P* < .001). A higher number of LDCT orders is apparent in men vs women and across racial and ethnic groups (Figure 1A) except for Native Hawaiian individuals, among whom there were slightly more LDCT orders for women. Differences in LDCT orders by men and women appeared when Asian, Native Hawaiian, and Pacific Islander racial and ethnic groups were disaggregated. Furthermore, in Figure 1B, Asian subgroups are represented, highlighting differences that emerge upon disaggregation into subgroups, especially among Chinese, Japanese, and Filipino men. Chinese and Filipino women were found to have the lowest percentage of LDCT orders (21% [7 participants] and 23% [23 participants], respectively) compared with other Asian female subgroups (Japanese, 34% [49 participants]; Korean, 55% [18 participants]).

**Figure 2.**
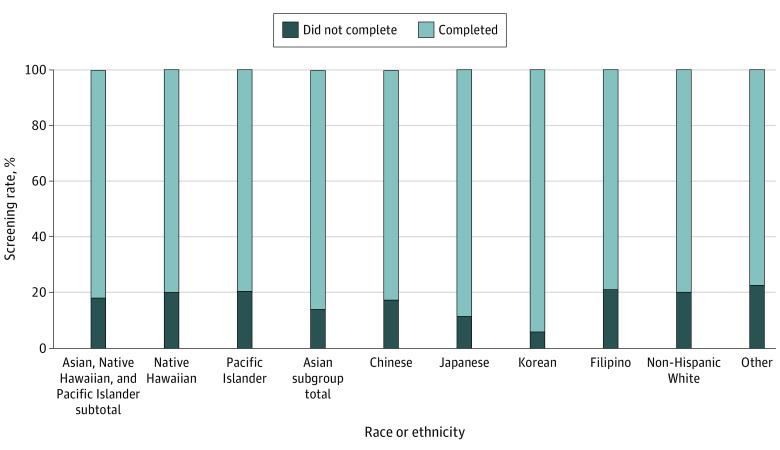
Low-Dose Computed Tomography Screening Completion Rates by Race and Ethnicity The other race and ethnicity category includes other Asian, African American, and American Indian or Alaska Native individuals.

A total of 838 of the 1030 members (81%) completed the LDCT test in the KPHI LCS program within 91 days from the order date (Figure 2). There was a 14% to 15% screening completion rate gap among groups. Asian individuals had the highest screening completion rate (266 participants [86%]); within Asian subgroups, Korean individuals had the highest rate (31 participants [94%]) followed by Japanese individuals (129 participants [88%]), Chinese individuals (28 participants [82%]), and Filipino individuals (78 participants [79%]). Native Hawaiian (149 participants [80%]) and non-Hispanic White (305 participants [80%]) individuals were next, followed by Pacific Islander (50 participants [79%]) and Hispanic (23 participants [79%]) individuals. The other race and ethnicity group had the lowest completion rate at 77% (68 participants). Screening completion rates by racial ethnic groups were not significantly different. Completion rates were not significantly different by sex.

Of the 54 LCS patients with a Lung-RADS stage 3 diagnosis, 50 (93%) completed a 6-month surveillance LDCT. Four LCS patients did not meet study-defined criteria for follow-up completion (2 LCS patients were followed-up after the 1-year window, 1 did not complete follow-up, and 1 moved to another state). Thirty-seven patients received a Lung-RADS stage 4 diagnosis, and 35 (97%) were followed-up with either a biopsy (17 patients [48%]), noninvasive procedure (16 patients [46%]), or cytological examination (ie, fine-needle aspiration) (2 patients [6%]) (2 LCS patients did not complete follow-up). Lung-RADS 4 follow-up procedures resulted in 13 malignant lesions identified (7 non–small cell carcinoma, 1 small cell carcinoma, 4 carcinoma [none other specified], and 1 mesothelioma) and 22 with benign findings.

## Discussion

The overall LCS completion rate was 81% in this multiethnic population; Korean and Japanese subgroups had the highest screening completion rates (94% and 88%, respectively), and the other race and ethnicity group had the lowest rate (77%). Although we observed that screening completion rates were not significantly different, there was a 14% to 15% screening completion gap between the Korean subgroup (94%) and the Filipino (79%), non-Hispanic White (80%), and Pacific Islander (79%) groups. These disaggregated race and ethnicity group completion rates indicate potential differences that should be explored in a larger sample.

A study^15^ conducted in an integrated safety-net health care system reported the LCS completion rate to be 67%, which is low compared with our study and other LCS trials in Europe^23^ and the US^24^ reporting 90% to 95% completion rates. However, a 67% screen completion rate may represent what might be observed in a real-world clinical setting. Factors associated with lower screening rates could be attributed to clinician variability in ordering tests; however, a previous study^15^ determined that this was not a contributing factor to differences in patients completing an LDCT. Lower income levels, no insurance, lack of a shared decision-making component in the LCS process, longer distance to screening site, and physician specialty or fewer years of experience also contribute to lower screening rates.^16^ Although these factors may be associated with screening completion rates, they are beyond the scope of this article and should be further examined among these racial and ethnic groups.

We did not find a significant difference in completion rates by sex and by race and ethnicity; however, there were differences by LDCT orders. More men vs women across racial and ethnic groups had an LDCT that was considered a proxy to eligibility in the KPHI LCS program. More men than women (60% vs 40%) were referred to the KPHI LCS program by their physician, which increased the pool of potentially eligible men. In addition, women may not readily meet eligibility criteria. Women receive diagnoses at younger ages^25^ and tend to have a lighter smoking history compared with non-Hispanic White men.^26^ Lung cancer rates are high among Chinese women; however, they have a lower prevalence of smoking and, therefore, may not qualify for eligibility into a LCS program.^3^ In our study, Chinese and Filipino women were found to have the lowest percentage of LDCT orders (21% and 23%, respectively) compared with other Asian female subgroups (Japanese, 34%; Korean, 55%). This may be because some women do not meet the eligibility criteria, resulting in a missed opportunity to identify and start preventive care. Fewer eligible women is one of the reasons for the proposed changes to the revised 2021 US Preventive Services Task Force criteria.^26^ In March of 2021, the US Preventive Services Task Force updated the guidelines to include individuals aged 50 to 80 years with a 20 pack-year history. Future studies should examine LCS completion and follow-up rates in these same racial and ethnic groups and by sex using these new guidelines that may help promote equity in screening eligibility for women.

Disaggregation of the Native Hawaiian and Pacific Islander groups and Asian subgroups provides insight into LCS behaviors that could be associated with many factors, including culture, immigration history, and nativity, that make these groups unique.^3^ In a study by Lee et al,^27^ aggregate colorectal screening rates for Asian American and Pacific Islander individuals (46.8%) were initially lower than those for non-Hispanic White individuals (57.7%); however, different rates appeared after disaggregation among Asian subgroups, where Japanese individuals had higher colorectal screening rates (59.8%) and Korean individuals had the lowest (32.7%). According to the Centers for Disease Control and Prevention Behavioral Risk Factor Surveillance System,^28^ Hawaii colorectal cancer screening rates were shown to vary whether Asian American and Pacific Islander individuals are aggregated, separated, and with subgroups examined. These differences may also be attributed to socioeconomic factors, including poverty and a lack of health insurance or a usual source of care. Social and behavioral factors associated with LCS participation among racial and ethnic groups and by sex should be further examined among disaggregated racial and ethnic groups to identify target groups and areas for intervention.

To our knowledge, this is the first study to report LCS completion rates among Native Hawaiian and Pacific Islander individuals, who experience high lung cancer incidence and mortality in both men and women. Our work also shows the variation in screening eligible rates across racial and ethnic groups among men and women upon disaggregation. In addition, we report high LCS completion (81%) and follow-up rates (93% for Lung-RADS stage 3 and 97% for Lung-RADS stage 4) within a diverse population and health care system that uses a Nurse Navigator as a part of clinical care. Patient navigation programs have been reported to increase breast, colorectal, prostate, and cervical cancer screening rates of vulnerable populations; however, few studies have examined navigation within LCS programs. A 2018 systematic review^29^ highlighted 4 LCS studies, where 1 study reported higher screening uptake and 3 studies noted a reduction in time to treatment.

### Limitations

We were unable to test for associations related to completion and follow-up rates for racial and ethnic groups by sex because of small numbers. In addition, the small sample size made it difficult to fully examine racial and ethnic differences and diagnostic outcomes. This would be important to explore in a larger sample because there have been racial and ethnic differences reported in extent or stage of disease.^3^ This study was also conducted in one geographical area; however, it also provided the opportunity to examine LCS in Hawaii, where 70% of the population are minoritized racial and ethnic groups.^30^

## Conclusions

In this study, LCS completion rates varied among Asian subgroups and Native Hawaiian and Pacific Islander groups. Disaggregation of these racial and ethnic groups is needed in future research involving larger samples to validate our findings and allow for a deeper dive into the social and behavioral factors that may be associated with LCS completion rates. This would inform the work toward mitigating racial and ethnic disparities that may exist in LCS and provide specific guidance to the care delivery system in developing targeted, culturally sensitive interventions. Furthermore, a Nurse Navigator may play an integral role in supporting LCS completion and follow-up rates through strengthening the continuity of care, resulting in faster initiation of preventive care and treatment. This may serve as a model for other systems, optimizing LCS completion and follow-up among vulnerable and at-risk populations.
