# Error in Figure 1

**DOI:** 10.1001/jamanetworkopen.2023.35206

**Published:** 2023-09-13

In the Original Investigation titled “Lung Cancer Screening by Race and Ethnicity in an Integrated Health System in Hawaii,”^1^ published January 20, 2022, the y-axis labels in Figure 1 should have read, “LDCT orders, %.” This article has been corrected.^1^

## References

[1] Oshiro CES, Frankland TB, Mor J, . Lung cancer screening by race and ethnicity in an integrated health system in Hawaii. JAMA Netw Open. 2022;5(1):e2144381. doi:10.1001/jamanetworkopen.2021.44381 35050353 PMC8777569

